# The identification of goat peroxiredoxin-5 and the evaluation and enhancement of its stability by nanoparticle formation

**DOI:** 10.1038/srep24467

**Published:** 2016-04-14

**Authors:** Xiaozhou Feng, Juanjuan Liu, Shuai Fan, Fan Liu, Yadong Li, Yuanyuan Jin, Liping Bai, Zhaoyong Yang

**Affiliations:** 1 Institute of Medicinal Biotechnology, Chinese Academy of Medical Sciences and Peking Union Medical College, Beijing, People’s Republic of China

## Abstract

An anticancer bioactive peptide (ACBP), goat peroxiredoxin-5 (gPRDX5), was identified from goat-spleen extract after immunizing the goat with gastric cancer-cell lysate. Its amino acid sequence was determined by employing 2D nano-LC-ESI-LTQ-Orbitrap MS/MS combined with Mascot database search in the goat subset of the Uniprot database. The recombinant gPRDX5 protein was acquired by heterogeneous expression in *Escherichia coli.* Subsequently, the anti-cancer bioactivity of the peptide was measured by several kinds of tumor cells. The results indicated that the gPRDX5 was a good anti-cancer candidate, especially for killing B16 cells. However, the peptide was found to be unstable without modification with pharmaceutical excipients, which would be a hurdle for future medicinal application. In order to overcome this problem and find an effective way to evaluate the gPRDX5, nanoparticle formation, which has been widely used in drug delivery because of its steadiness in application, less side-effects and enhancement of drug accumulation in target issues, was used here to address the issues. In this work, the gPRDX5 was dispersed into nanoparticles before delivered to B16 cells. By the nanotechnological method, the gPRDX5 was stabilized by a fast and accurate procedure, which suggests a promising way for screening the peptide for further possible medicinal applications.

Cancers have caught the whole world’s attention for its morbidity and mortality. According to the GLOBOCAN estimates, about 14.1 million new cancer cases and 8.2 million deaths occurred in 2012 worldwide^1^. However, the routine treatment by surgery in combination with palliative chemotherapy does not bring out expected clinical efficacy due to their side-effects^2^,^3^. In recent years, more and more novel treatment strategies, such as targeted therapy and immunotherapy, have been applied to treat patients with advanced cancers^4^. For instance, the programmed death-1 (PD-1) protein interacts with ligands on tumor and immune cells in the tumor micro-environment and undermines the corresponding antitumor immunity^5^.

Originated from various biological processes, bioactive proteins/peptides have shown low toxicity as anticancer therapeutic agents, especially compared with the classical chemotherapeutics, and thus have drawn much attention from cancer researchers^6^,^7^. Besides, bioactive proteins/peptides can also help enhance the efficacy of chemotherapy and reduce toxicity in clinical treatments. The anticancer bioactive peptide (ACBP), investigated in this work, was first identified by Su *et al.* from goat spleen extract after immunizing the goat with gastric cancer lysates, and it exhibited anti-neoplastic effects in *in vitro* experiments and low toxicity in long-term animal experiments^8^,^9^,^10^,^11^,^12^,^13^.

However, it is still not known how the protein exerts its anticancer bioactivity effectively in physiological conditions. Moreover, the complex procedure of the time-consuming immunization of goat with gastric cancer lysate and the low yield of peptide extraction from spleen extract hinders further investigation on its biological modulations and clinical use as an anticancer agent. So, it is urgently needed to identify the amino acid sequence of gPRDX5 to get insight into its functional mechanism and further applications.

The Nano-flow Liquid Chromatography in tandem with Orbitrap Mass Spectrum offers a good way for authentication purposes^14^,^15^. With the technology, Yang *et al.* screened gelatinous Chinese medicines, such as Asini Corii Colla, Cervi Cornus Colla and Testudinis Carapacis ET Plastri Colla, which were extensively used as both hemopoietic and hemostatic agents to treat vertigo, palpitation, hematuria, and insomnia in traditional Chinese medicine clinics and consumed as a popular tonic for valetudinarians. Based on enzymatic digestion, a new protocol was established to identify protein complex with natural products of the herb medicines for authentication purposes^16^. Recombinant DNA technology has allowed the production of proteins with anticancer activity by employing expression vectors as those used in bacteria or yeast expression systems^17^. So, it is significant to identify the amino acid sequence of gPRDX5 and enrich the protein by heterogeneous expression for further investigations.

Because of their poor stability, high variability, short half-lives and high molecular weight, the proteins/peptides are more sensitive to the change of enzymatic environment and pH than conventional drugs, and it is also difficult for them to penetrate the intestinal mucosa by gastrointestinal administration. All these characteristics contribute to their limited medicinal application currently. Nanotechnology has witnessed a rapid development in pharmaceutical area in recent years because of its effectiveness as a drug delivery method and its unique biological properties in encapsulating or confining small molecule anticancer drug to avoid interaction with healthy cells and preferentially accumulate in the tumors through the Enhanced Permeability and Retention (EPR) effect^18^. Scientists have applied nanoparticles (NPs) for cancer treatments with great succcess. The NPs can easily accumulate inside tumor tissues and have higher retention rates. Furthermore, NPs used to deliver therapeutic protein/peptide drugs can not only overcome biological barriers and reduce side effects, but also can improve drug stability, solubility and bioavailability^19^,^20^,^21^,^22^,^23^,^24^. Meanwhile, the bioactivity of NPs is easier to be estimated for their stability. They exhibit better sensitivity towards cancer cells and are thus expected to be more effective in cancer treatment. The screening of anticancer bioactive proteins/peptides in combination with nanotechnology promises an effective method for medicinal purposes.

## Results and Discussion

### The sequencing of gPRDX5

The gPRDX5 has attracted scientific interest in recent years since it was first identified by Su *et al*^10^. It could be a highly potential anticancer biological product for inducing cell apoptosis and cell cycle arrest^11^. However, entangled in natural protein mixture of goat spleen extract, it was difficult to purify, thus not quantitatively available to make in-depth study of its anticancer mechanism and promote its medicinal applications. So, to overcome the problem, it’s crucial to sequence the target protein in the first phase, and acquiring the protein through heterogeneous expression would be the subsequent prerequisite for further investigation. LC-MS/MS Spectrometry was chosen for the identification, quantification and detailed primary structural analysis of the target peptide from the extracted protein mixture.

**Figure 1:**
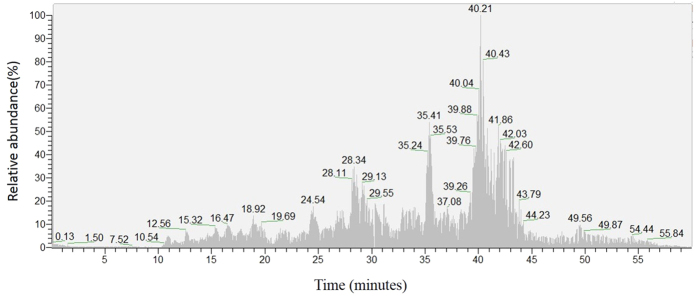
NanoLC-orbitrap MSD TIC of ACBP’s digests. NanoLC-orbitrap MSD, nano-flow liquid chromatography in tandem with orbitrap mass spectrum detection; TIC, total ion chromatogram.

The sequencing procedure was performed, with the fragment information of 240 different peptides acquired in our MS measurements, as shown in Fig. 1. The information of the individual fragment was analyzed by a Mascot database search into the mammalia subset of the Uniprot database. Eight fragments corresponding to a special protein scored as high as 110.75, and thus were analyzed first. Totally eight different fragments were eluted from capillary column between 20 to 50 minutes. The exact mass of the peptide ions was calculated according to the precursor mass and isotope pattern displayed in the MS spectrum at every time point, and the MS and MS/MS fragments obtained were all subject to *de novo* sequencing for further analysis by Xcalibur 2.0.7. By this method, the eight fragments identified from the digests of the peptide were exclusively selected for determination, as summarized in Table 1. All the selected fragments were within the range of 100–2000 m/z of multiple charges at 2+ or 3+, which were composed of 8–23 amino acid residues. Among these fragments, lysine was always at the C-terminal site of the sequences. When the ion was 2+, Xcorr ≥ 2.2 and when the ion was 3+, the Xcorr ≥ 3.75. These results showed that the peptide’s information was credible.

All of the sequences were compared with other known proteins based on an online Basic Local Alignment Search Tool (BLAST) analysis, available on the National Center for Biotechnology Information (NCBI) website (http://blast.ncbi.nlm.nih.gov/Blast.cgi), and were used as input sequences for a Position-Specific Iterated BLAST (PSI-BLAST) search program against the non-redundant protein sequences databases (nrdb). As summarized in Table 1, the highest sequence identity (73.46%) was found in the partial sequences of peroxiredoxin-5 from Ovis aries, which comprises of 162 amino acids. So we speculated that the Peroxiredoxin-5 from goat may be the target peptide with the potential to kill tumor cells as mentioned in previous studies^8^,^10^,^13^. Subsequently, the corresponding gene DNA sequence was acquired with the blasted information of the potential peptide.

### The heterogeneous expression of gPRDX5

**Figure 2:**
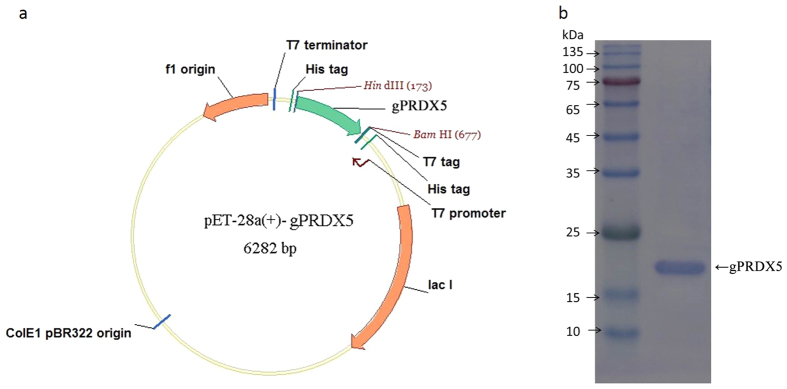
The construction of gPRDX5-pET-28a(+) plasmid and the acquisition of heterogeneous expressed gPRDX5 protein. (**a**) The DNA fragment of gPRDX5 was connected to the expression vector pET-28a(+) and the plasmid was then transformed to BL21 (DE3) for protein purification, (**b**) The gPRDX5 was acquired by heterogeneous expression and it molecular weight was at about 18 kDa.

The degenerate primers were designed as Sense:ATCCATGAAATTGTTTTGGATACGC and Anti-sense:CATTCTGTAGTGGCACTCCTGGC, and the goat genome DNA was used as PCR template. Eventually, the targeted gene DNA fragment was acquired successfully and it was 486 bps in length. Subsequently, the gene DNA was re-amplified by PCR with restriction sites of *Bam*HI and *Hind*III. After the digestion of the DNA fragment and vector pET-28a(+) by the two enzymes, a recombinant plasmid, pET28a(+)-gPRDX5, was constructed (Fig. 2a), and was then transformed to *E. coli* BL21 (DE3) for the expression and purification of the peptide. Finally, a pure protein of about 18 KD was acquired by the heterogeneous expression (Fig. 2b), comparable to the theoretical molecular-weight value at 17.3 KD. The heterogeneous expression of the protein solved the crucial problem of low yield and complex procedure by goat-spleen extraction, therefore making it easier to study the mechanism of the gPRDX5.

### Preliminary screening of gPRDX5 as an anticancer reagent by CCK-8 assay

**Figure 3:**
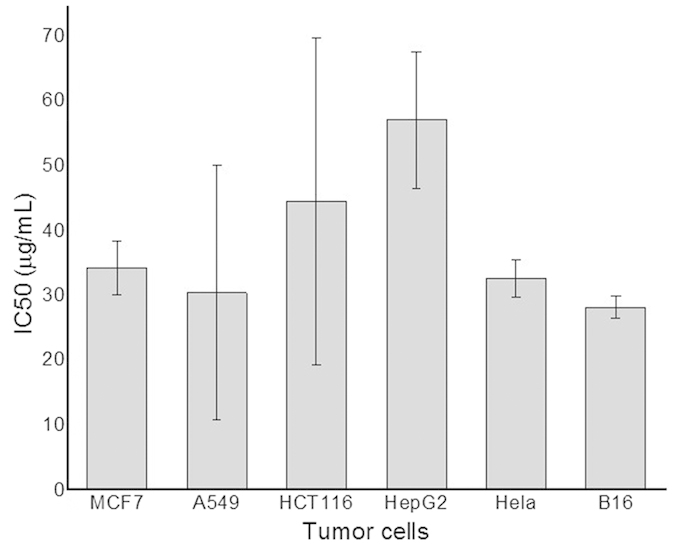
The preliminary screening of the anticancer bioactivity of gPRDX5. The gPRDX5 was sensitive to all the six tumor cell lines with the IC50 values at about 34.15, 30.326, 44.47, 56.985, 32.545 and 28.107 for MCF7, A549, HCT116, Hep G2, Hela and B16 cells, respectively (*P* < 0.05). The sensitivity was the highest against B16 cells. The values varied from sample to sample within the same tumor cells.

The gPRDX5 was first analyzed by BLAST, http://blast.ncbi.nlm.nih.gov/Blast.cgi, and the results showed that its homogeneity to the peroxiredoxins V (prxV) of human being was up to 89%. It was reported that prxV was a candidate breast tumor marker of population specificity^25^, and was also essential for the protection against apoptosis in human lung carcinoma cells^26^, which gave us confidence to extrapolate that the gPRDX5 might be a potential anticancer reagent with a wide spectrum. Therefore, its *in vitro* anti-tumor activity was evaluated by the tumor cell lines of human breast cancer cell line: MCF-7, human alveolar basal epithelial cell line: A549, human colon cancer cell line: HCT116, human liver hepatocellular cell line: HepG2, human immortal cell line: Hela and mouse melanoma cell line: B16. As revealed by the CCK-8 assay, the gPRDX5 exhibited rather high cytotoxicity against all the six tested tumor cell lines, suggesting that it possessed broad anti-cancer activities, (Fig. 3) with IC50 ranging from 28 μg/mL to 57 μg/mL in our assessment.

The gPRDX5 showed the highest anti-cancer activity against B16 cells. However, it was noteworthy that the IC50 values of gPRDX5 fluctuated greatly among the parallel CCK-8 assays. Given that the gPRDX5 is easy to denature even when kept at 4 °C, the fluctuation of IC50 values in Fig. 3 might be due to its instability. Hence, in order to accurately evaluate the anti-tumor activity of the gPRDX5, it was vitally necessary to improve its stability for further investigations and applications.

### Preparation and Characterization of gPRDX5 Nanoparticles (NPs)

Protein/peptide-based drug formulations are commonly prepared as solid dosage forms for treatment delivery because they are more stable than those in liquid state^27^. In pharmaceutical industry, freeze drying is the most popular method to lyophilize proteins and peptides to increase the stability^28^. The major advantages of the nanotechnology application in pharmaceutical industry^29^ are the efficacy increment on drug administration, side effects decrement and stability enhancement concerned about unstable drugs. Thus, the nanoparticle formation technology is employed here for our investigation.

To avoid the disadvantage of the conventional rapid and uncontrolled crystal growth leading to over-sized particles, the gPRDX5 was dispersed into separated emulsion droplets of oil-in-water (O/W) emulsion^30^. The emulsion was then frozen rapidly by exposure to liquid nitrogen, and went through subsequent cryodesiccation. Along with this procedure, the protein molecules encapsulated inside the same emulsion droplet transformed into nano-crystallite individually. The final size of gPRDX5-loaded nanoparticles could be well controlled by adjusting the protein concentration in the oil phase and supersonic vibration intensity during the emulsion preparation.

The surfactants such as Cremophor® EL and Pluronic® F-127 (F-127) can maintain the thermal stability of proteins/peptides after freeze drying. Besides, the F-127 has been approved by the US Food and Drug Administration for intravenous use, so we used the amphiphilic tri-block copolymer F-127 as a surfactant to stabilize the oil-water interface. After removal of both the oil and water phase by cryodesiccation, the F-127 encapsulated gPRDX5 in the inner core of nanoparticles consisting of hydrophobic poly(propyleneglycol) (PPG) block, with poly(ethyleneglycol) (PEG) block as hydrophilic outer surface. The F-127 prevented the gPRDX5 from aggregation and preserved its biological activity as well, similar to the role of β-cyclodextrin in maintaining the biological activity of insulin^31^. The improved stability of gPRDX5-loaded NPs using F-127 as a stabilizer revealed the promising *in vivo* delivery of gPRDX5 *via* inhalation and injections.

**Figure 4:**
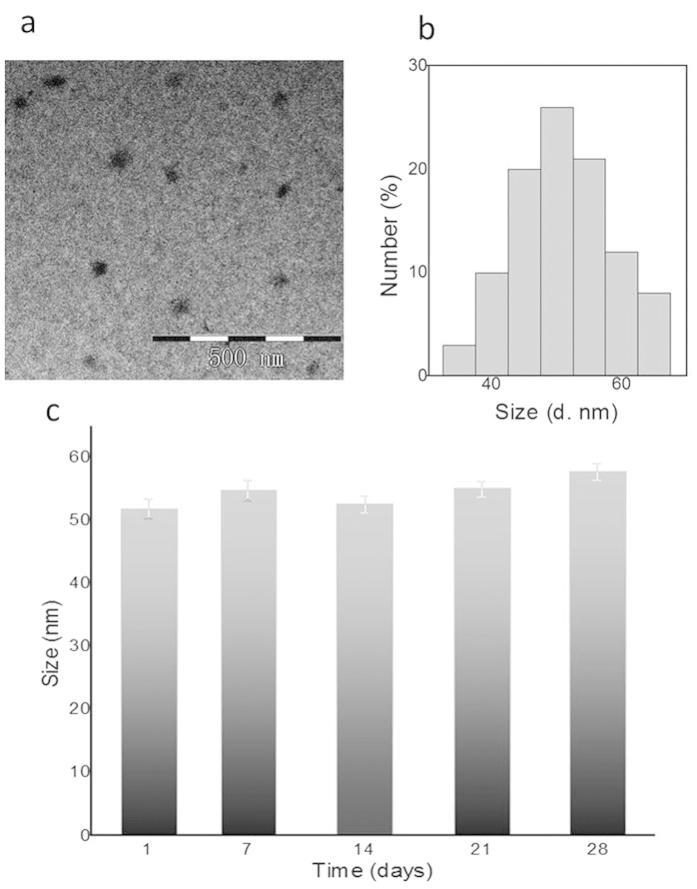
Characterizations of gPRDX5 nanoparticles. (**a**) The typical gPRDX5 NPs by TEM with the diameters ranging from 30 to 70 nm. (**b**) The average size was at about 50 nm. (**c**) The NPs remained stable in size within a month.

The Fig. 4a exhibits the morphology of gPRDX5 NPs, displaying a plain solid spheroid-like structure. The size distribution of gPRDX5 NPs is shown in Fig. 4b, with an average size of about 51 nm (Fig. 4c). The yield of gPRDX5 NPs could be up to 90%, and the NPs were found to be stable without aggregation for over four weeks. These results confirmed that the protein nanoparticles were well-decorated and stable enough for further study.

### Cellular uptake and *in vitro* anti-tumor activities of gPRDX5-NPs

The undecorated proteins/peptides normally have difficulty getting into cancer cells when they are administered. With the development of nanotechnology, more and more nanoparticles have been used to enhance the stability and efficacy of protein drugs. Chitosan NPs, developed firstly in the 90 s, have been widely studied for the delivery of proteins and antigens^32^.

Normally the particle size of NPs is an important parameter affecting the cellular uptake rate and amount significantly. The submicron-size particles are taken up more efficiently than larger size microparticles. Maurizio *et al.* reported that NPs with smaller size might be more advantageous in anticancer treatment^33^.

**Figure 5:**
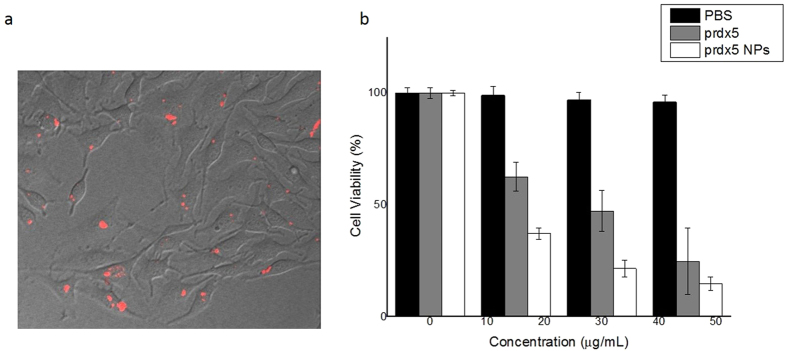
The anticancer effect of gPRDX5-NPs on the B16 cells. (**a**) The CLSM images of the internalized NPs in B16 cells. (**b**) The cytotoxicity of PBS, gPRDX5 and gPRDX5-NPs (*P* < 0.05). The gPRDX5-NPs was more sensitive against the B16 cells.

Hence, we assumed that incorporating the gPRDX5 into nano-size particles, with the approximate size at about 51 nm, might significantly enhance the corresponding cellular uptake. By employment of Confoaser Scanning Microscopy (CLSM), the cellular uptake of gPRDX5 NPs was evaluated in B16 cells. As shown in Fig. 5a, the gPRDX5 NPs were extensively internalized by B16 cells, suggesting that encapsulating gPRDX5 into nanoparticles greatly enhanced the intracellular accumulation of gPRDX5.

Given that the adequate intracellular accumulation is the first prerequisite for a certain drug to exert bioactivity, we subsequently investigated the *in vitro* antitumor activities of gPRDX5 NPs in B16 cells by CCK-8 assay (Fig. 5b), using PBS and gPRDX5 as controls. Results in Fig. 5b showed that gPRDX5 NPs showed much higher cytotoxicity against B16 cells than the gPRDX5 alone, indicating that incorporating gPRDX5 into nanoparticles is an efficient strategy to improve the bioactivities of gPRDX5. We assumed that the elevated anti-tumor activity of gPRDX5 NPs might be due to the increased cellular uptake and the enhanced stability of gPRDX5 NPs.

The results obtained here suggested that the gPRDX5 might be a promising anticancer drug candidate, and also confirmed that nanoparticles could enhance the stability and the subsequent bioactivity of protein drugs. Therefore, nanotechnology can be a meaningful tool for the final successful clinical application of heat-sensitive macromolecular drugs, including vaccines, proteins, peptides and nucleic acids.

## Conclusion

In summary, first of all, we determined the sequence information of the target peptide by 2D nano-LC-ESI-LTQ-Orbitrap MS/MS, and with the information we determined the target peptide as goat peroxiredoxin-5 (gPRDX5). The pure gPRDX5 was then acquired by heterogeneous expression in BL21(DE3) to overcome the problem of its difficult extraction from natural tissues. Secondly, we preliminarily screened the anticancer bioactivity of the protein in MCF-7, A549, HCT116, HepG2, Hela and B16 cells by CCK-8 assay and found that the gPRDX5 was sensitive to all six tumor cell-lines, especially to the B16 cells. However, its instability made it hard to evaluate its anticancer capability efficiently. It is well-known that the nanotechnology is a good way to stabilize protein/peptide, so we developed a new protocol to screen the peptide’s anticancer performance. Thirdly, we successfully acquired gPRDX5 nanoparticles by using oil-in-water (O/W) technology with the help of F-127, and subsequently evaluated the anticancer capability of the NPs by CCK-8 assay. The results suggested that the gPRDX5 NPs were more stable than the peptide alone, and the resulted NPs still had the ability to kill cancer cells. The nanotechnology is a good promising way to stabilize unstable bioactive proteins/peptides for the investigation of their modulating mechanisms and the possible medicinal applications in clinical treatments. All these suggestions taken together indicate that our protocol for identifying and modifying targeted protein/peptide with pharmaceutical excipients is a feasible strategy and is an effective modality for the screenings of other bioactive proteins/peptides.

## Methods

### Preparation of protein sample

The protein sample containing the target peptide was kindly provided by Professor Xiulan Su, Clinical Medicine Research Center, Inner Mongolia Medical College, the People’s Republic of China.

One hundred milligrams of the sample was dissolved in 10 mL Milli-Q water by vortex and sonication and then centrifuged at 12,000 rpm at 4 °C for 30 minutes. The supernatant was collected for subsequent membrane ultrafiltration by centrifugal filter unit (5 kDa molecular weight cut-off; EMD Millipore) at 3,000 rpm at 4 °C, according to the manufacturer’s instructions, to remove most of the small molecules in the solution. The remaining concentrated brown sample solution in the upper vessel of the unit was completely transferred to another filter unit (10 kDa molecular weight cut-off; EMD Millipore) for concentration and the supernatant solution was again transferred to a glass container and subjected to further lyophilization, resulting in a pale and light block mass which was then stored in a desiccator at room temperature for following experiments.

Ten milligrams of the lyophilized mixture was dissolved in 2.0 mL of 50 mM TES buffer (0.36 mM CaCl_2_, pH 7.4) to a final concentration of 5.0 mg/mL for trypsase digestion. After sonication and vortexing to promote dissolution, some solid material still remained and the solution was subject to centrifugation at 12,000 rpm for 30 minutes. Then, 100 μL of supernatant was denatured at 95 °C for 10 minutes and cooled down to room temperature, and 2.0 μL of trypsase solution (0.10 mg/mL in TES buffer) was added for incubation at 37 °C for over 24 hours before the full digestion was finished. The digestion was fulfilled thoroughly for one time with the addition of excessive enzyme, and the completeness of the digestion was ensured by SDS–PAGE with silver staining.

### 2D nano-LC-ESI-LTQ-Orbitrap MS/MS

The trypsase-digested peptides were analyzed by Nano LC-MS/MS on an Orbitrap Elite mass spectrometer (Thermo Fisher Scientific, Waltham, MA, USA), coupled with a HPLC system from Thermo Fisher Scientific. The spraying was done using a Michrom’s Thermo Captive Spray nanoelectrospray ion source at a capillary temperature of 250 °C and 1.5 kV source voltage. The separation was performed in a reverse-phase Acclaim PepMap RSL column (75 μm internal diameter  × 15 cm, 2 μm particles), also from Thermo Fisher Scientific, which was maintained at 35 °C throughout the analysis. The separation was carried out at a flow rate of 200 nL/min, using 0.1% aqueous formic acid solution as eluent A and 0.1% formic acid in 90% aqueous acetonitrile solution as eluent B. The 60-minute gradient elution started at 3% of eluent B for 1 minute, followed by 3%–35% of eluent B for 47 minutes, increased to 50% of eluent B within 4 minutes, again increased to 80% within 0.1 min, and kept the concentration for 1.3 minutes. Subsequently, the mobile phase was turned back to 3% of eluent B within 0.1 minute and maintained for 6.5 minutes for equilibrium.

Data acquisition was done in positive ion mode using the LTQ Tune Plus software (Thermo Fisher Scientific), alternating between full MS (100–20,000 m/z, resolution 60,000, with 1 μscan per spectrum) and FT-MS/MS (100–2,000 m/z, resolution 15,000, with 1 μscan per spectrum) for the ten most intense ions (with a 500-count threshold). Fragmentation was performed in high-energy collisional dissociation mode at 32% normalized collision energy. Automatic gain control target for both full MS and FT-MS/MS was set to 1e + 06, and the precursor ion-charged state screening was activated. Dynamic exclusion list was enabled with an exclusion duration of 30 seconds and an exclusion list size of 500.

The whole sequences of the fragments were first analyzed by Mascot, and then the *de novo* sequencing of each peptide was conducted by PEAKS software automatically. Local confidence was the confidence (presented as a percentage) that a particular amino acid was present in the *de novo* peptide at a particular position.

### DNA sequencing and recombinant-plasmid construction

The degenerate primer was designed to get the corresponding DNA sequence of the target peptide. The procedure was directed by the website: https://icodehop.cphi.washington.edu/i-codehop-context/Welcome. The PCR DNA products were digested by enzymes *Bam*HI and *Hind*III and then connected to linearized pET-28a (+) vector. The recombinant plasmid was amplified, then transformed into *E.coli* BL21 (DE3) for protein expression.

### Protein expression and purification

Recombinant *E. coli* BL21 (DE3) bearing the gPRDX5 plasmid was cultivated at 37 °C overnight in LB medium supplemented with 50 μg/mL kanamycin. 1 mL culture was reinoculated into 100 mL of LB media. When the culture reached an OD_600_ of 0.6–0.8, IPTG was added in to a final concentration of 0.1 mM, and the culture was cultivated at 16 °C with shaking at 200 rpm for 12 h. The culture was harvested by centrifugation at 4 °C and 2500 × g for 10 min. After the harvested bacteria pellet was lysed and the lysate was centrifuged, the supernatant sample was loaded onto a Ni-NTA agarose gel column pre-equilibrated with PBS buffer (50 mM sodium phosphate, 0.5 mM NaCl, pH 7.4) containing 10 mM imidazole. The elution was made by a linear imidazole gradient (10–400 mM) with the same PBS buffer. The purity and molecular weight of the proteins were confirmed by the sodium-dodecyl-sulfate polyacrylamide gel electrophoresis (SDS-PAGE, 10% polyacrylamide).

### Fabrication and characterization of goat peroxiredoxin-5-loaded nanoparticles

The gPRDX5 peptide-loaded nanoparticles (gPRDX5 NPs) were prepared *via* oil-in-water emulsion freeze-drying method, which generally is a three-step process. The first step was the emulsion preparation. The oil phase (O) was dichloromethane solution containing 40 mg/mL gPRDX5. An aqueous solution containing 1% Pluronic® F-127-COOH was the water phase (W). The volume ratio of oil phase to water phase was fixed at 1:20, and the mixture was crashed by ultrasonic (100 w, 10 min) to obtain emulsion (O/W), the whole process was carried out on ice. Secondly, the above emulsion was further added into 20-fold the volume of the water phase, and the mixture was frozen by liquid nitrogen followed by lyophilisation using a vacuum freeze-dryer. Finally, the NPs suspension was obtained when the lyophilized product was dispersed into water and the free surfactant was removed by ultracentrifugation (800,000 rcf, 10 min). The morphology of gPRDX5-NPs was observed by using transmission electron microscope (TEM, JEM-1400, JEOL). The size and zeta potential of gPRDX5-NPs were determined by Zeta Sizer (Nano Series, Malvern), respectively.

### Cell culture and *in vitro* anti-tumor activities of gPRDX5 NPs

Cell lines of MCF-7, A549, HCT116, HepG2, Hela and B16 were all purchased from American Type Culture Collection (ATCC). All of these cells were cultured in RPMI-1640 culture medium (Invitrogen, USA) supplemented with 10% heat-inactivated fetal bovine serum (FBS), 100 U/mL penicillin, and 100 U/mL streptomycin, and maintained in a humidified atmosphere of 5% CO_2_ at 37 °C. The cells in logarithmic growth phase were used to conduct all cell experiments in this study. The *in vitro* anti-tumor activities of gPRDX5 in above cell lines were evaluated *via* Cell Counting Kit-8 assay (CCK-8, Dojindo. Molecular Technologies, Inc., Kumamoto, Japan). All 6 cell lines were respectively seeded into 96-well plates in RPMI-1640 containing 10% FBS at densities of ~1–3 × 10^3^ cells per well. Then, the cells were cultured overnight for attachment. The blanks were prepared by only adding culture medium. Subsequently, in various test groups, the media containing various concentrations of gPRDX5 or gPRDX5 NPs were added. The cells without the addition of gPRDX5 NPs were taken as control group. Six replicates were conducted for each group. After 4 days culture, 10 μL of CCK-8 assay reagent was added into each well to incubate the cells at 37 °C for 2 h. Finally, the optical density value was determined at 450 nm using microplate reader (Bio-Rad Laboratories Inc., Tokyo, Japan). The BL16 cells were used to evaluate the gPRDX5 NPs and the method was the same as previously mentioned. Inhibition rate of cell proliferation was calculated according to the following equation.


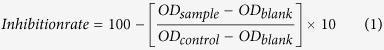


### Cellular uptake of gPRDX5 NPs

For CLMS visualization, B16 cells were seeded into a 24-well plate at a density of 1 × 10^5^ cells/well with sterile glass slides and incubated for 24 h to allow attachment. Then, the previous medium was replaced by the one containing gPRDX5 NPs. After 24 h incubation, the medium was removed, and the cells were washed with cold PBS (pH 7.2) followed by fixing with 4% paraformaldehyde and staining with cell membrane dye Cy5.5. The fluorescence images of cell membrane and gPRDX5 NPs were captured by CLSM at 650 nm with a UV/vis spectrophotometer (Emax Precision Microplate Reader, Molecular Devices, Sunnyvale, CA, USA).

### Statistical analysis

Statistical analyses were conducted by using GraphPad Prism, Origin 9.0 and Microsoft Excel. Measurement uncertainties throughout are denoted by error bars and shading as indicated in the figure legends. All statistical tests were two-tailed with testing level thresholds of α = 0.05.

## Additional Information

**How to cite this article**: Feng, X. *et al.* The identification of goat peroxiredoxin-5 and the evaluation and enhancement of its stability by nanoparticle formation. *Sci. Rep.* **6**, 24467; doi: 10.1038/srep24467 (2016).

## Figures and Tables

**Table 1:** List of the possible fragments of gPRDX5.

Sequence	NanoLC	Orbitrap MSD		PEAKS	
	*t*_R_(min)	Precursor MH^2+^****		Mass error	
ETDLLLDDSLVFLFGNHR	47.58	1052.54114	2104.07500	1.88 ppm	5.11
THLPGFVEQAGALK	25.73	489.93610	1467.79374	−1.21 ppm	4.43
GVLFGLPGAFTPGCSK	37.31	804.41742	1607.82756	1.52 ppm	4.19
VGDAIPSVEVFEKEPGNK	27.05	957.99225	1914.97722	−1.89 ppm	4.13
FSmVIEDGIVK	24.78	627.32526	1253.64324	−1.15 ppm	3.37
LLADPNGTFGK	21.84	566.80414	1132.60100	1.09 ppm	3.20
FSMVIEDGIVK	31.24	619.32855	1237.64983	0.05 ppm	2.87
VNLAELFK	34.97	467.27441	933.54155	1.19 ppm	2.85
SLNVEPDGTGLTCTLAPNILSQL	40.89	1207.12024	2413.23320	2.15 ppm	2.85

Abbreviations: MSD, mass spectrum detection; NanoLC, nano-flow liquid chromatography; tR, retention time.
